# CXCR5+ CD8 T Cells: Protective or Pathogenic?

**DOI:** 10.3389/fimmu.2019.01322

**Published:** 2019-06-18

**Authors:** Kristen M. Valentine, Katrina K. Hoyer

**Affiliations:** ^1^Quantitative and Systems Biology Graduate Program, University of California, Merced, Merced, CA, United States; ^2^Department of Molecular Cell Biology, School of Natural Sciences, University of California, Merced, Merced, CA, United States

**Keywords:** B cell follicle, germinal center, chronic viral infection, cancer, autoimmune disease, CD8 T cells

## Abstract

CD8 T cells are infrequently considered part of germinal center reactions. Yet, a distinct CXCR5+ CD8 T cell subset identified within the B cell follicle and germinal center in situations of chronic antigen has recently been defined. CXCR5+ CD8 T cells maintain transcriptional and phenotypic features consistent with the CD8 T cell nomenclature of a non-exhausted, effector memory population. CD8 T cell localization to the B cell follicle suggests a functional profile similar to CD4 T follicular helper cells that are licensed to promote B cell responses. The functional mechanisms defined under different immune settings, while largely similar, differentially control disease pathogenesis. CXCR5+ CD8 T cells control viral load during infection, and also promote antibody-mediated autoimmune disease progression. The existence of this novel CXCR5+ CD8 T cell subset in human and murine models of disease may provide a paradigm shift in our understanding of germinal center reactions.

## Introduction

**Figure 1:**
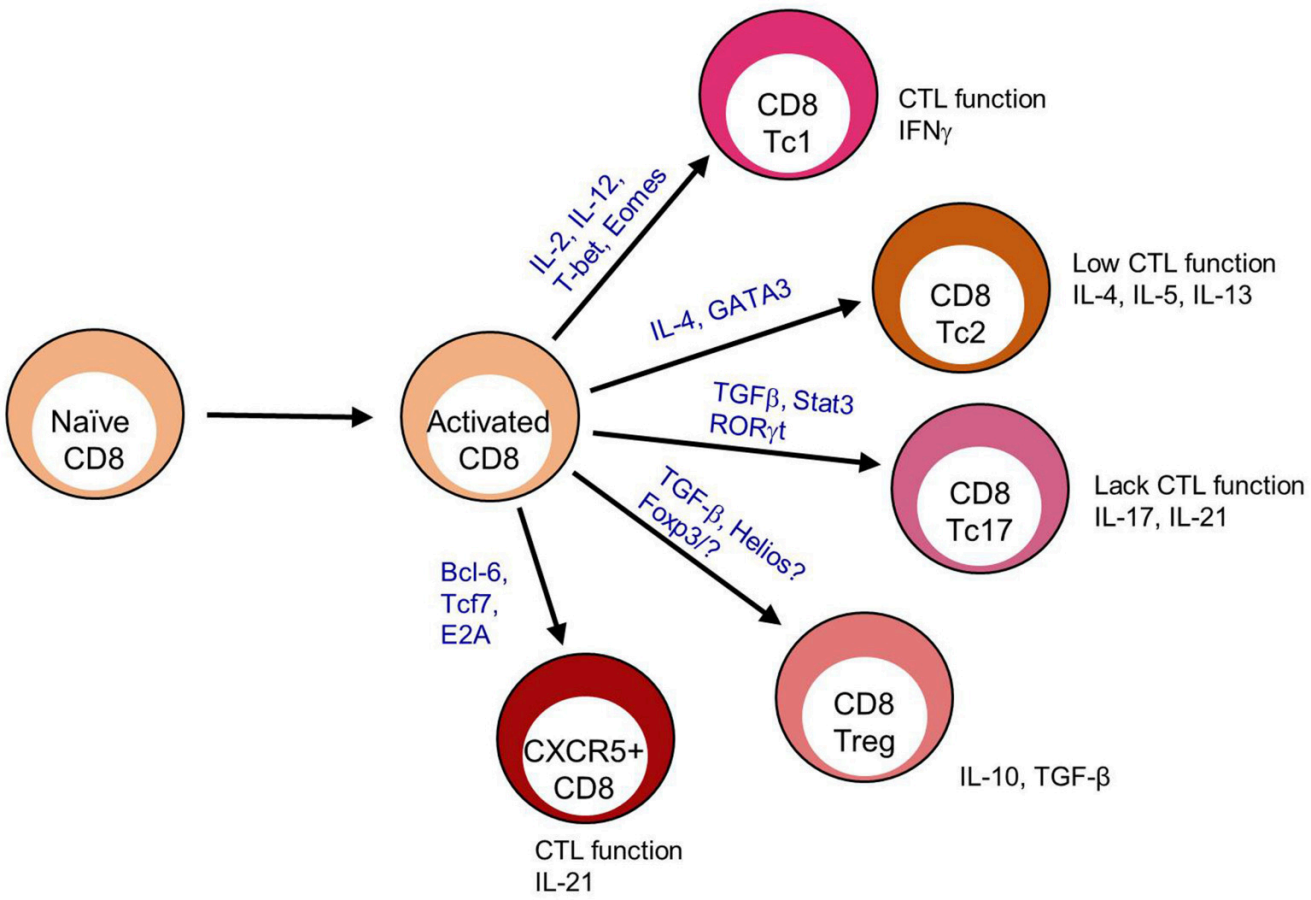
CD8 T cell differentiation subsets. After antigen recognition, activated CD8 T cells that receive specific TCR interactions, cytokine signaling, and dendritic cell signals upregulate transcription factors that program terminal differentiation outcomes including CD8 T cytotoxic (Tc) 1, Tc2. Tc17, Treg, and CXCR5+ subsets.

As CD8 T cells have been identified both phenotypically and functionally into distinct subsets beyond that of the classical cytotoxic CD8 T cells (CTL), it follows that novel CD8 T cell subsets may yet still emerge. A recent focus on CD8 T cells has highlighted a diversity of functional responses. Like CD4 T cells, CD8 T cells differentiate into multiple subsets that are customized to a specific infection and immune settings (1, 2) (Figure 1). CD8 Tc1 cells comprise the canonical CTL subset, producing IFNγ, perforin, and granzymes involved in targeted cell killing (3). CD8 Tc1 cells arise predominately in response to viral and intracellular infections but also in some autoimmune diseases to induce pathogenic tissue destruction. CD8 Tc2 cells are implicated in response to specific allergens and typically exhibit reduced CTL function and produce IL-4 and IL-5. CD8 T regulatory cells (Tregs) identified in the context of self-reactive responses are less well-defined and may have multiple phenotypes (4, 5). Some CD8 Tregs localize to the B cell zone but are also found in circulation (6–8). Beyond these effector subsets, at least three memory CD8 T cell types [T effector memory (Tem), tissue resident memory, and central memory] have been extensively described (9).

This review characterizes a novel subset of CXCR5+ CD8 T cells capable of infiltrating the B cell follicle in settings of chronic antigen exposure and inflammation. CXCR5+ CD8 T cells maintain an independent phenotype from their CXCR5- CD8 T cell counterparts. Their functional role largely depends on the immune setting, yet they maintain a cytotoxic capacity that aids in the control of viral infection, tumor growth inhibition, or the promotion of inflammation and autoimmune responses. Finally, CXCR5+ CD8 T cells have a unique developmental profile utilizing genes similar to both CD4 T follicular helper (Tfh) cell development and CD8 effector memory or memory-stem cell differentiation. Some of the gene variation found in CXCR5+ CD8 T cells seems to be dependent upon the conditions under which these cells arise.

## A Novel T Cell Subset: CXCR5+ CD8 T Cells

CXCR5+ CD8 T cells develop under several conditions of chronic antigen and inflammation. They are transcriptionally and phenotypically distinct from other CD8 T cell subsets. Most studies find that CXCR5+ CD8 T cells gain entry into the B cell follicle. Yet, there is no clear consensus defining the function of this CXCR5+ CD8 T cell subset.

### A Distinct Phenotype

Transcriptional and phenotypic profiling, in combination with a variety of functional responses, indicate several possible classifications for CXCR5+ CD8 T cells including: cytolytic, Tem/stem cell, exhausted and follicular helper CD8 T cells. The differences leading to these classifications likely depend on the particular immune setting and the subsequent functional responsiveness of CXCR5+ CD8 T cells.

**Figure 2:**
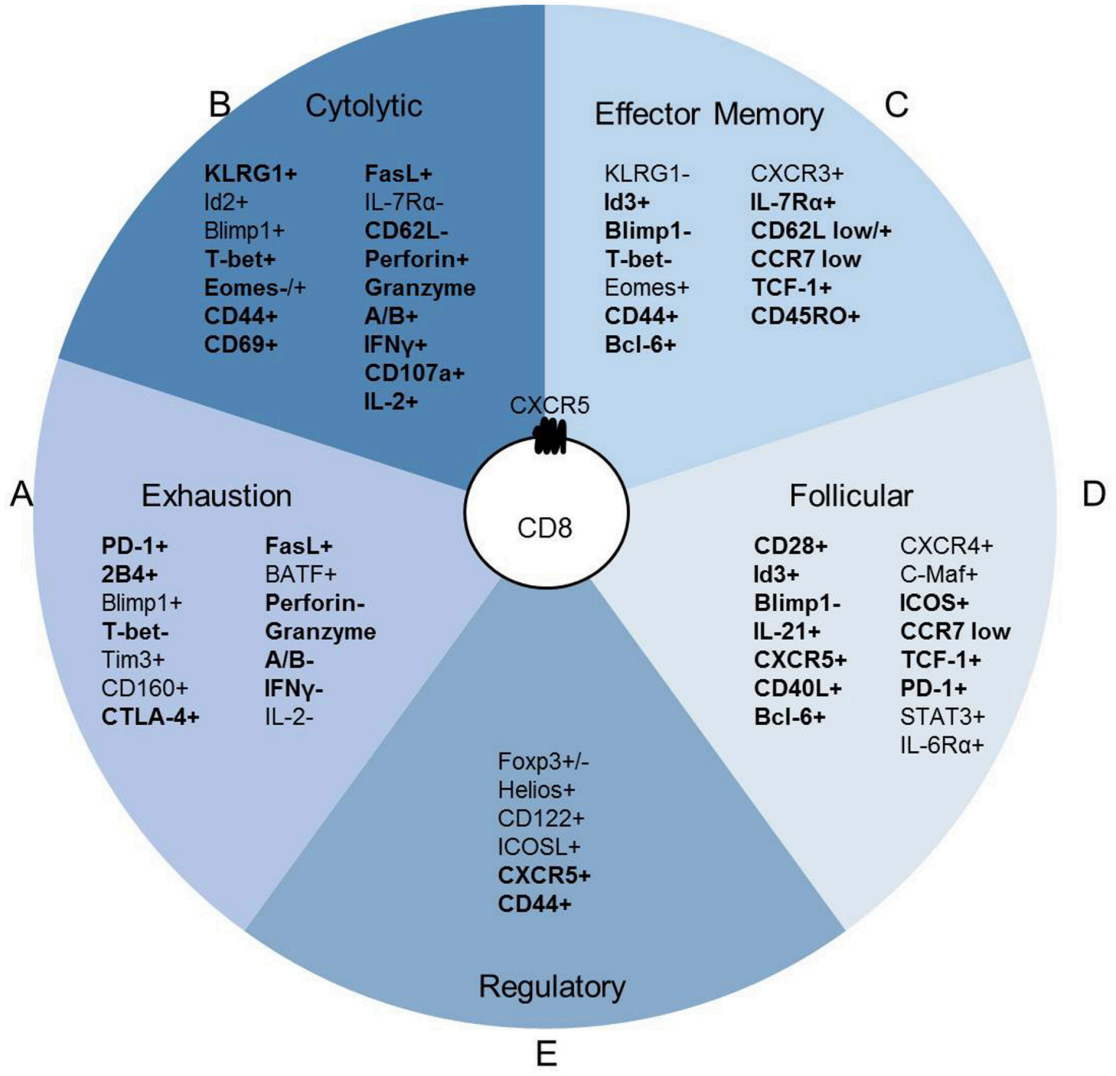
CXCR5+ CD8 T cells maintain a distinct expression pattern. CXCR5+ CD8 T cell protein expression relative to T cell subsets; **(A)** CD8 T cell exhaustion (10), **(B)** CD8 cytotoxic T cell, **(C)** CD8 T effector memory (Tem) (9), **(D)** CD4 T follicular helper (Tfh) (18), and **(E)** CD8 T regulatory cell (Treg) (4). Bold indicates literature confirmed protein expression in CXCR5+ CD8 T cells.

During chronic viral infections CD8 T cells are frequently associated with an exhausted profile including reductions in IL-2 production, cytolytic function and proliferation. This shift toward exhaustion is associated with increased expression of the co-inhibitory molecules PD-1, 2B4, Tim3, KLRG1, CD160, and Lag3 among others (10). When CXCR5+ CD8 T cells wereevaluated for an exhausted phenotype, gene expression profiles reveal that CXCR5+ CD8 T cells have increased *pdcd1* (PD-1) expression but reduced *faslg, ctla4, lag3, havcr2* (Tim-3), and *cd244* (2B4) in some studies (11–14). While in other studies, CXCR5+ CD8 T cells express elevated PD-1 and FasL with variable CTLA-4, Lag3 and Tim-3, but reduced 2B4 expression (12, 13, 15–17) (Figure 2A). Cytolytic functionality as measured by granzyme B, perforin, and CD107a, provides a mixed picture for CXCR5+ CD8 T cells as a non-exhausted population. CXCR5+ CD8 T cells express decreased *grzma, grzmb*, and *prf1* gene expression when compared to CXCR5- CD8 T cells in viral infection (12). Yet, tumor-infiltrating and virus-specific CXCR5+ CD8 T cells appear to maintain cytolytic capacity upon *ex vivo* stimulation (13, 17, 25). However, considering the variability in exhaustion marker expression as well and the maintenance of cytolytic capacity (described in section II of this review), CXCR5+ CD8 T cells are likely not functionally exhausted. Specifically, CXCR5+ CD8 T cells express elevated KLRG1, CD44, T-bet, and Blimp-1 compared to CXCR5- and naïve CD8 T cells indicative of an activated, fully differentiated cytolytic subset (12, 13, 15) (Figure 2B).

**Table:** 

**Cytolytic**	KLRG1+ (15), T-bet+ (12, 19), Eomes- (19), CD44+ (12, 13, 15), CD69+ (20), FasL+ (20), CD62L- (12, 20), Perforin+ (19, 21–24), Granzyme A+ (20, 23), Granzyme B+ (16, 19, 21, 23–26), IFNγ+ (12, 14, 15, 20, 22, 25, 27, 28), CD107a+ (14–16, 22, 23), IL-2+ (12, 14, 20).
**Exhaustion**	PD-1+ (12, 13, 16, 17, 22, 24, 29, 30), 2B4+ (17), T-bet- (25), CTLA-4+ (14), FasL+ (20), Perforin- (13, 14, 20, 26, 27, 31), Granzyme A- (13, 27), Granzyme B- (12–14, 22), IFNγ- (13, 17).
**Effector Memory**	Id3+ (13), B limp1- (13, 25), T-bet- (25), CD44+ (12, 13, 15), Bcl6+ (12–14, 25, 29), IL-7Rα+ (12, 13, 20), CD62L- (12, 20), CD62L+ (13), CCR7- (13, 17, 20, 21, 24), TCF-1+ (12, 13), CD45RO+ (19, 20).
**Follicular**	CD28+ (12, 14, 20), Id3+ (13), Blimp1- (13, 25), IL-21+ (28, 29), CD40L+ (29), Bcl6+ (12–14, 25, 29), ICOS+ (12, 13, 19, 22), CCR7- (13, 17, 20, 21, 24), TCF-1+ (12, 13), PD-1+ (12, 13, 16, 17, 22, 24, 29, 30).
**Regulatory**	CD44+ (12, 13, 15)

While CXCR5+ CD8 T cells appear to maintain a cytolytic phenotype, this phenotype does not account for the upregulation of *cd127* (IL-7Rα), *tcf7* (TCF-1), *id3, eomes*, and *cd44* that are commonly associated with an effector memory phenotype (12, 13) (Figure 2C). Im et al. defined lymphocytic choriomeningitis virus (LCMV)-specific CXCR5+ CD8 T cells as stem-like CD8 Tem that proliferated into both CXCR5+ and CXCR5- CD8 T cell subsets (12). Similarly, CXCR5+ CD8 T cells isolated from PBMCs of cancer patients proliferate more than CXCR5- CD8 T cells after TCR stimulation (16, 25). Leong et al. defined CXCR5+ CD8 T cells in LCMV infection as an effector memory-like (CD62L+ IL-7R+) population by RNA sequencing (13). Perhaps, most convincingly, in simian immunodeficiency virus (SIV) infection CXCR5+ CD8 T cells in comparison to SIV-specific CXCR5- CD8 T cells, and CD8 T cells under autoimmune conditions compared to naïve CD8 T cells express significantly more *bcl6* and less *prmd1* (Blimp-1) (14, 29). The Tem phenotypic description attributed to CXCR5+ CD8 T cells is probably indicative of the chronic antigen exposure under which these cells have thus far shown to arise.

Alternatively, although not completely counter to evidence of an effector memory subset, CXCR5+ CD8 T cells share a transcriptional profile similar to that of CD4 Tfh cells in SIV infection by RNA sequencing of virus specific CXCR5+ CD8 T cells (14). CXCR5 is most commonly associated with B cell zone migration and homing, and has been described extensively on B cells and CD4 Tfh cells (32). CXCR5+ CD8 T cells express costimulatory, transcription factors, inhibitory genes, and proteins similar to CD4 Tfh, including: increased *cd200, icos, cd28, bcl6, id3, ctla4, pdcd1*, and *faslg* and reduced *prdm1, id2*, and *havcr2* (Tim-3) (18) (Figure 2D). These data are supported by research in the inflammatory environment of human nasal polyps, in which a CXCR5+ CD8 T cell population arises and express FasL, CD28, OX-40, and ICOS post-*ex vivo* stimulation (20). In an autoimmune disease setting, CXCR5+ CD8 T cells express cytolytic molecules associated with canonical CD8 T cell function, but also express *cxcr5, icos, bcl6, pdcd1, cd40l*, and *il21* (29). In Hogdkin's lymphoma, CXCR5+ ICOS+ CD8 T cells are more closely related to CD4 Tfh and not other T cell subsets based on gene expression profiles (33).

A CD8 Treg population that maintains germinal center (GC) reactions and controls autoimmune disease has been described within the B cell follicle (7). CD8 Tregs can express FoxP3, or associate with the transcription factor Helios (4, 6). When identified as CXCR5+, CD8 Tregs express ICOSL, CD44, and CD122 (5, 7) (Figure 2E). He et al. reported that in LCMV infection, CXCR5+ CD8 T cells were ICOSL and Helios negative but CD44+ (15). Similarly, in the context of autoimmune disease, CXCR5+ CD8 T cells largely lack ICOSL, FoxP3, and Helios expression (unpublished data). It is possible that CXCR5+ CD8 T cells, in some situations, are CD8 Treg cells (8); but most reports suggest an effector phenotype for these cells. Together, transcriptional profiling and subsequent validation by flow cytometric analysis, identify a CXCR5+ CD8 T cell population with the potential to behave as cytotoxic canonical CD8 T cells, promote B cell responses and respond as CD8 Tem (Table 1).

**Table 1:** CXCR5+ CD8 T cells maintain antigen-specific transcription and localization.

	**Transcription**		**Location**	**Reported functional capacity**	
	**High**	**Low**			
SIV/HIV	*bcl6, cd28, cd40, cd83, cd200, ctla4, il2, irf4*	*cd244, grzma, grzmb, id2, runx3, Prdm1* (Blimp-1)	GC, follicle, and extrafollicular space of lymph node and spleen	Less cytotoxic effector CD8 T cell that controls infection	(13, 14, 21, 24, 31)
LCMV	*bcl6, cd200, icos, id3, il2, il7rα* (CD127), *sell* (CD62L), *tcf7* (TCF-1)	*cd244, fasl, grzma, grzmb, havcr2* (Tim-3), *id2, prdm1, prf1*	B cell follicle and T cell zone of splenic white pulp	Less cytotoxic effector CD8 T cell that controls infection and maintains proliferative capacity	(13, 15, 31)
Cancer	*bcl6, grzma, grzmb, ifnγ, il2, pdcd1, prdm1, prf1, tbx21 (T-bet), tnf*	*ctla4, havcr2* (Tim-3), *lag3*	Peripheral blood, tumor infiltrating, and tumor draining lymph node	Non-exhausted cytotoxic effector CD8 T cell that promotes tumor suppression	(11, 16, 23, 25, 30, 33)
Autoimmunity and inflammation	*bcl6, ccr7, cd200, ctla4, cxcr5, eomes, fasl, grzma, grzmb, havcr2, icos, ifng, il21, irf4, maf, pdcd1, prdm1, sh2d1a* (sap)^*^	Not tested	B cell follicle lymph node and spleen, ectopic GC in tissue- specific disease	Promotes autoimmune antibody responses	(20, 27, 29, 34, 35)

CXCR5+ CD8 T cells have been identified under at least four antigen conditions including SIV/HIV infection, LCMV infection, cancer, and autoimmunity/inflammatory.

* *Gene transcription compared WT CD8 T cells and IL-2 deficient CD8 T cells in autoimmunity. All other gene transcription was compared between CXCR5+ CD8 T cell populations and CXCR5- CD8 T cell populations*.

CXCR5+ CD8 T cells have been described in both humans and mice and may account for the variation in cytolytic capacity, homing, and function attributed to CXCR5+ CD8 T cells in multiple immunologic settings. Here we endeavor to summarize relevant data from humans and mice from multiple disease settings. As with CD4 Tfh cells, early descriptions of CXCR5+ CD8 T cells have stemmed from human samples (20, 32, 36) and augmented by transcription factor knockout and reporter mice (13, 15, 37). While multiple studies have characterized CXCR5+ CD8 T cells in humans and mice, (13, 15) CXCR5+ CD8 T cell development in chronic, but not acute settings promotes investigations primarily in human immunodeficiency virus (HIV) and SIV. Continued characterization of CXCR5+ CD8 T cells will require mechanistic studies better facilitated in mice. Describing CXCR5+ CD8 T cell transcriptional pathways using reporter and knockout mice (13, 15), evaluating CXCR5+ CD8 T cell population kinetics during disease across multiple organs (29), and evaluating the independent role of CXCR5+ CD8 T cells in controlling disease via animal transfers (12) are benefits of studies in mice. These findings once resolved with cross-species variation, will provide rationale designs for CXCR5+ CD8 T cells as therapeutic targets for human disease.

### Antigen-Specific CXCR5+ CD8 T Cell Responses and Localization

As the principal chemokine receptor that facilitates entry into the B cell zone, CXCR5 expression on CD8 T cells instigated investigation into CXCR5+ CD8 T cell homing. CD8 T cells, by CCR7 upregulation and not CXCR5 expression are excluded from the B cell follicle. However, of the total CD8 T cell population only the small frequency that upregulates CXCR5 during SIV infection localize in and around the follicle (21). In addition to observations in SIV infection, CXCR5 expression on human CD8 T cells in HIV is closely associated with proximity and responsiveness to CXCL13 in the lymph node (17, 20). CXCR5 expression on CD4 Tfh is required to migrate toward CXCL13 and facilitate GC development (38). Some CD4 Tfh developmental signals do not require B cell help initially to induce the CD4 Tfh transcription factor, Bcl6 (39, 40). However, once at the B-T border, CD4 Tfh cells interact with B cells to gain entry into the GC and solidify their transcriptional profile via Bcl6 using ICOS and PD-1 interactions (37, 41, 42). In murine LCMV, CXCR5+ CD8 T cells may also require B cell interactions to enter the follicle as CXCR5+ CD8 T cells that maintained *ccr7* gene expression retain their capacity to localize to the T cell zone and are excluded from the GC (12). A requirement for B-T cell interaction has yet to be directly investigated in CXCR5+ CD8 T cell development and function.

**Figure 3:**
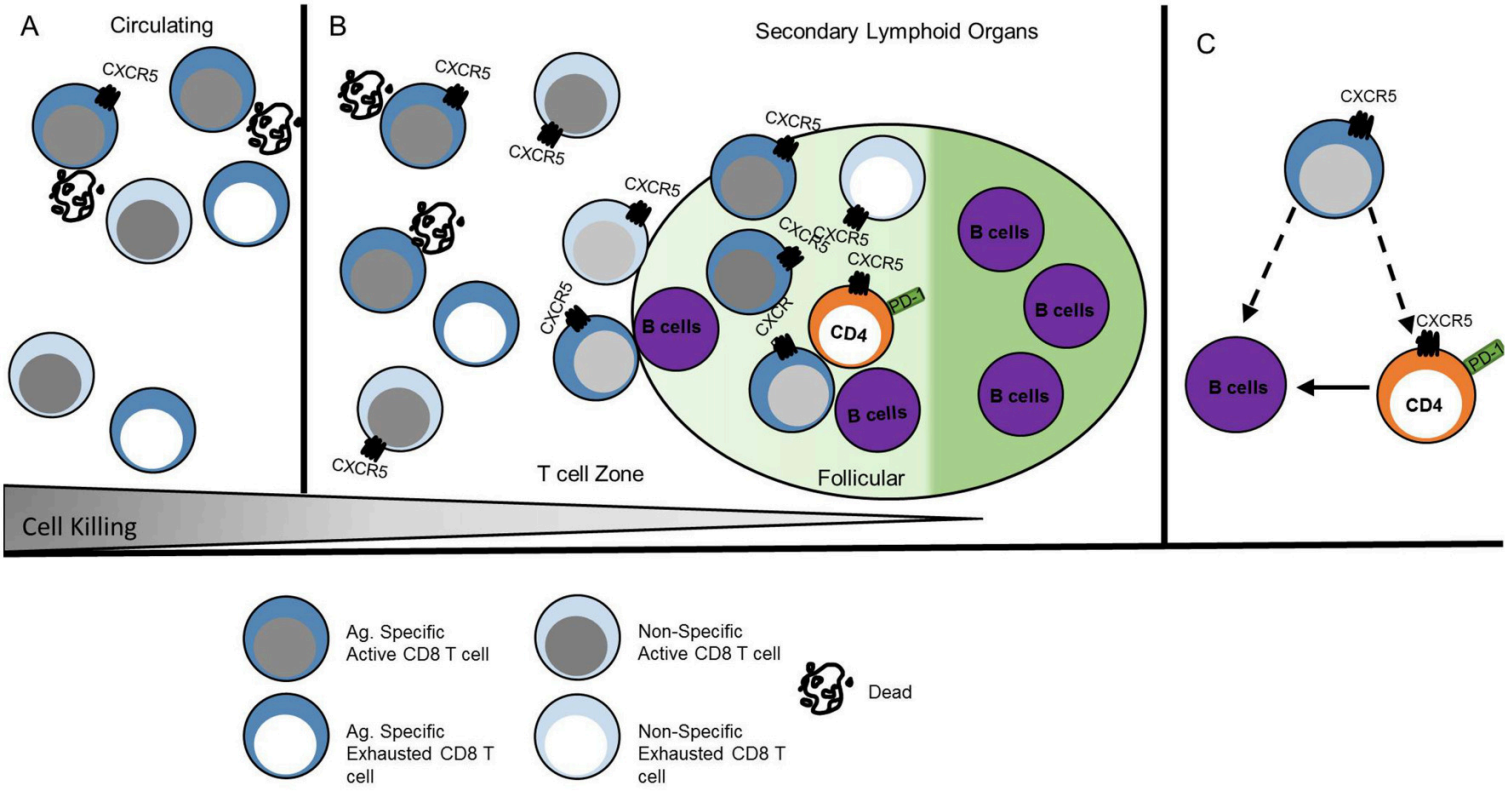
CXCR5+ CD8 T cell function is anatomically instructed. **(A)** Antigen-specific CXCR5+ CD8 T cells in peripheral blood circulation induce direct cell killing, whereas other CD8 T cells may be functionally exhausted. **(B)** CXCR5+ CD8 T cells within secondary lymphoid organs that localize to the extrafollicular space maintain higher direct cell killing capacity than CXCR5+ CD8 T cells within the follicle. Antigen-specific CXCR5+ CD8 T cells are maintained at similar frequency in the extrafollicular space and follicle. **(C)** Follicular CXCR5+ CD8 T cells maintain the capacity for cytotoxicity; different antigen scenarios likely elicit various functional outcomes that promote direct B cell or CD4 Tfh cell interactions. Dark blue cells indicate antigen specific T cells. Dashed lines indicate potential interactions.

CXCR5+ CD8 T cell accumulation in the follicle does not appear to be dictated by antigen concentration or immune setting but rather by conditions of chronic inflammation and immune activation (17, 20, 29). In chronic infections, both high and low viral load correlate with expanded CXCR5+ CD8 T cell populations throughout the follicle, including the GC (21, 24, 43). Chronic HIV, SIV and LCMV infection studies identified antigen-specific CXCR5+ CD8 T cells (12–15, 31). Peripheral blood isolated HIV-specific CD8 T cells are more cytolytic than lymphoid CD8 T cell populations. Additionally, within the lymphoid CD8 T cell populations, CXCR5+ CD8 T cells maintain a robust cytolytic phenotype compared to CXCR5- CD8 T cells (19). In models of chronic viral infections, LCMV-specific CXCR5+ CD8 T cells identified within the extrafollicular space and germinal center also display a cytolytic phenotype compared to CXCR5- CD8 T cells (12, 15, 22, 31). CXCR5- CD8 T cells are likely exhausted (12, 13, 15, 19) which may account for the described increases in CXCR5+ CD8 T cell cytotoxicity that is higher than CXCR5- CD8 T cells but lower than peripheral blood CD8 T cell populations, at least in humans. Thus, irrespective of viral infection and host species, CXCR5+ CD8 T cells maintain cytolytic capacity in both the blood and lymph node in humans and mice (Figure 3A). The localization or direct cell killing capacity of CXCR5+ CD8 T cells requires continued investigation.

Interaction time and specific signals during GC interactions may redirect the transcription of CXCR5+ CD8 T cells and alter effector functions as described for CD4 Tfh cells (44). In HIV infection, some CXCR5+ CD8 T cells demonstrate high lytic potential in GC regardless of antigen specificity (17), while other antigen-specific CXCR5+ CD8 T cells are less lytic in patients without strong immune responses (22). The frequency of CXCR5+ CD8 T cells isolated from pancreatic and colorectal cancer tumor masses correlate with improved patient outcomes suggestive of tumor control (11, 30). In humans at least, viral-specific or tumor infiltrating CXCR5+ CD8 T cells likely utilize cytolytic mechanisms to control viral infection and tumor growth in secondary lymphoid organs, ectopic GC, and the tumor microenvironment. The presence of antigen-specific CD8 T cells in the extrafollicular space and within the follicle suggests that CXCR5+ CD8 T cells directly interact with virally infected CD4 Tfh cells. The potential for cytolytic responses may explain the negative correlation observed between the frequency of CXCR5+ CD8 T cells and reduced CD4 Tfh cell frequency and viral load (13, 14, 17, 19, 24) (Figure 3B).

In the context of chronic inflammation and autoimmune disease, CXCR5+ CD8 T cells likely employ diverse mechanisms to promote inflammatory responses and advance disease pathogenesis at the site of autoreactive responses within ectopic GCs or lymphoid tissue. Influenza-specific murine CD8 T cells migrate to lung ectopic GCs and interact with B cells following intranasal infection (16, 45). CD40L+ CD8 T cells within human synovial fluid, that are likely antigen-specific for joint proteins, are required for the formation and maintenance of ectopic GCs in rheumatoid arthritis inflammation (27, 34). Murine autoimmune CXCR5+PD-1^hi^ CD8 T cells expressing CD40L and GL-7 promote antibody responses (29). CXCR5+ CD8 T cells in human nasal polyps that localize to B cells promote inflammatory damage (35). Together, these data reveal a pattern of CXCR5+ CD8 T cell homing related to antigen accumulation and the site of local immunological responses (Figure 3C).

## CXCR5+ CD8 T Cell Function

### Viral Infection

CXCR5+ CD8 T cells have been predominately explored in the context of chronic viral infections. In chronic LCMV and SIV infection, CXCR5+ CD8 T cell frequency inversely correlates with viral load and associates with a reduction in virus-producing cells attributing cytolytic function to CXCR5+ CD8 T cells (13, 15, 21, 24, 31). However, in similar studies of chronic SIV and LCMV infection, CXCR5+ CD8 T cells display reduced cytolytic protein expression coupled with a more stem-like effector memory phenotype (12).

There are a number of possible explanations for differences in cytolytic activity across the existing viral CXCR5+ CD8 T cell literature (46) including the comparison across species of CXCR5+ CD8 T cell populations, population heterogeneity, differentiation states and subsequent cellular interactions potentially dependent on the disease model. Immerging evidence indicates an effector memory-like CD8 T cell population, that develops in situations of chronic antigen and cell exhaustion, with the capacity for cytolytic function. Inhibitory receptors such as PD-1, frequently used to describe exhausted CD8 T cells, may also denote a follicular-helper like subset of CD8 T cells that maintains its cytotoxic effector function and elicits GC entry (17, 22). Adoptively transferred LCMV-specific CXCR5+ CD8 T cells rapidly expanded to reseed the exhausted CXCR5- CD8 T cell niche (12) and significantly reduced viral load following PD-1 blockade (15). Yet, during short PD-1 blockade treatments in HIV infection, a PD-1+ subset of CXCR5+ CD8 T cells, instead produced less TNFα and IFNγ cytokines (22). Within the CXCR5+ population, there likely exists multiple effector functions similar to differences observed in CD4 Tfh cell function as it relates to PD-1 expression, follicular localization, and terminal differentiation.

In addition to a cytolytic role in controlling infection, direct interactions with infected B cells and CD4 Tfh cells may also facilitate CD8 T helper-like functions in the follicle. Human CXCR5+ CD8 T cells from chronic hepatitis B viral (HBV) infection produce IFNγ and influence IgG and IgA production when co-cultured with naïve B cells or memory B cells (28). CD8 T cells infiltrate influenza infected lungs and promote IL-21 dependent antibody class-switching and prolonged B cell survival (45). This follicular helper type function may also act to promote a tissue specific antiviral response on CD4 Tfh cells differing from the cytolytic response facilitated by chronic viral infection reservoirs in secondary lymphoid organs.

### Cancer

Cancer represents a situation of chronic, low-level self-antigen much like the situation induced by chronic viral infection. In B cell lymphoma-bearing mice and diffuse large B cell lymphoma patients, CXCR5+ CD8 T cells likely arise to directly target cancer cells (13, 23). Whereas, in HBV-related hepatocellular carcinoma, viral responses may initially induce CXCR5+ CD8 T cells that then target cancer cells (16). In colorectal and pancreatic cancer, CXCR5+ CD8 T cells arise and respond to cancer cells (11, 25, 30) suggesting a prevalent role for chronic antigen exposure in the development of tumor-specific CXCR5+ CD8 T cells.

CXCR5+ CD8 T cells isolated during immune responses to cancer maintain cytolytic potential toward tumor cells despite protein expression typically indicative of exhaustion. Circulating CXCR5+ CD8 T cells isolated from patients with HBV-related hepatocellular carcinoma and diffuse large B cell lymphoma expressed granzyme B and CD107a that likely contributed to tumor cell and B cell lysis (16). Circulating, tumor infiltrating, and lymphoid CXCR5+ CD8 T cells also express PD-1 and Tim-3 but are functionally less exhausted than CXCR5- CD8 T cells (11, 16). Yet, combined blockade of Tim-3 and PD-1 augment CXCR5+ CD8 T cell specific lysis of tumor cell targets indicating reduced lytic potential (16, 30). Further, CXCR5+ CD8 T cells in colorectal cancer maintain a cytolytic capacity to directly lyse tumor cells but can also influence B cell secretion of IgG, suggesting multiple mechanisms for tumor control by these cells (25).

In spite of the fairly robust cytolytic potential and activity by CXCR5+ CD8 T cells, tumor cells likely employ inhibitory mechanisms to suppress CXCR5+ CD8 T cell function. *In vitro* neutralization of IL-10 or IL-10R pathway improved granzyme A, granzyme B, and perforin-mediated cytotoxicity by CXCR5+ CD8 T cells (23). IL-10 or PD-1L blockade induced CXCR5+ CD8 T cell targeted specific cell lysis of autologous tumor cells (16). Enhancing specific cell lysis by preventing tumor suppression of CXCR5+ CD8 T cells or by improved CXCR5+ CD8 T cell function provides a new potential target for existing cancer therapeutics. As pancreatic and colorectal cancer disease-free survival time is positively correlated with CXCR5+ CD8 T cell frequency (30), the maintenance of a CXCR5+ CD8 T cell population may prolong cancer treatment efficacy.

### Inflammation and Autoimmune Disease

The mechanisms by which CD8 T cells mediate autoimmune disease pathology remain largely unresolved, but inflammation and autoimmune disease studies suggest a helper function for CXCR5+ CD8 T cells. In the absence of CD8 T cells, GC formation is prevented in rheumatoid arthritis and disease is delayed in spontaneous auto-antibody mediated disease (27, 29). Differential synovial ectopic GC formation is associated with CD8 T cell recruitment in a CD40L dependent manner (34), and follicular dendritic cells could not be retained in synovial GCs grafted into NOD-SCID mice in the absence of CD8 T cells (27).

While CD40L CD8 T cells appear to have a role in mediating ectopic GC formation, they do not produce the cytolytic proteins perforin and granzyme A, but maintain expression of IFNγ and TNFα (27). CXCR5+ CD8 T cells identified in human tonsils express IFNγ, TNFα, granzyme A, and IL-2 (20). Human tonsil CD8 T cells co-cultured with B cells promoted B cell survival like that of CXCR5+ CD4 T cells and induced IgG class-switching (20). IL-21-producing CD8 T cells from human nasal polyps co-express IFNγ and IL-21 to induce B cell class-switch to IgG when co-cultured with B cells (35). IFNγ is a known mediator of B class-switch to IgG2a/c, yet CXCR5+ CD8 T cells that arise in spontaneous autoimmune disease induced B cell class-switch to predominately IgG1. When transferred into TCRα deficient mice, IL-2-deficient CD8 T cells alone did not induce B cell differentiation or class-switching. Instead, CD8 T cells together with CD4 T cells enhanced plasma cell differentiation and induced IgG1 and IgG2b (29).

The primary location of CD8 T: B cell interactions within the GC, mantle zone, or extrafollicular foci, and, whether the mechanisms promoting antibody class-switch are via direct contact or secreted cytokines are predominately unexplored. Although yet untested, CXCR5+ CD8 T cell function in autoimmune disease likely includes canonical cytotoxic mechanisms in addition to acquired Tfh mechanisms. In contrast to most chronic viral infections and cancer, autoimmune and inflammatory CXCR5+ CD8 T cells likely promote the disease state, although the mechanisms that alter or advance GC reactions, in addition to direct cell lysis, may be similar.

## Distinct Developmental Pathways for CXCR5+ CD8 T Cells

**Figure 4:**
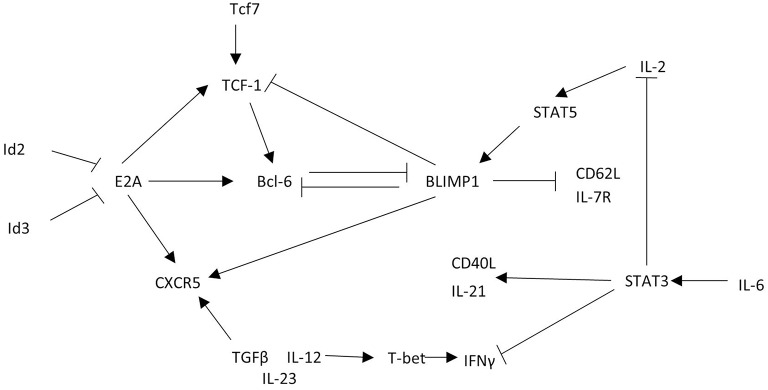
CXCR5+ CD8 T cells acquire a unique transcriptional profile from other CD8 T cells. CXCR5+ CD8 T cells express the transcription factor Bcl6 that is maintained by TCF-1 and E2A. This transcriptional interaction allows for CXCR5 upregulation and expression of functional proteins. Arrows indicate promoting interactions. Blunted lines indicate inhibitory interactions.

The similarity of CXCR5+ CD8 T cell phenotype and function to other CD8 T cell subsets described in Figure 2 likely provide overlapping, if not identical models, for differentiation and function of these cells described in other excellent reviews (9, 10, 47). Here we propose a transcriptional network that explains the gene expression and function observed in CXCR5+ CD8 T cells (Figure 4).

To explore CXCR5+ CD8 T cell regulation, the *cxcr5* promoter has been evaluated by chromatin immunoprecipitation deep sequencing. In response to a chronic viral infection the *cxcr5* promoter in CXCR5+ CD8 T cell contains two Blimp-1 binding sites and one E2A binding site, in addition to binding sites at the *bcl6* and *tcf7* promoter similar to that of CD8 effector memory T cells and CD4 Tfh cells (13, 15, 48). Retroviral Bcl6 induction of LCMV-specific donor cells increased CXCR5+ CD8 T cell frequency with a corresponding TCF-1 upregulation and Blimp-1 repression (13), suggesting a regulatory connection between TCF-1 and Bcl6 antagonism of Blimp-1 expression (39, 49). Further, CXCR5+ CD8 T cells do not arise in the absence of *tcf7* (TCF-1) during LCMV infection (12, 13). Experiments to test the significance of Blimp-1 regulation in CXCR5+ CD8 T cells preferentially expand from cells deficient in Blimp-1 using mixed chimeras of Blimp-1 deficient and WT bone marrow indicating that, like CD4 Tfh cells, CXCR5 expression is, in part, suppressed by Blimp-1 mediated transcription (13).

Id2 and Id3 regulate E2A and other e-family proteins responsible for regulating gene transcription in CD4 T cells (48, 50). E2A overexpression enhances CXCR5 expression increasing cytotoxic responses via CD107a expression and PD-1 downregulation, producing a less exhausted phenotype (15). In CXCR5+ CD8 T cells Id2 is downregulated and Id3 is upregulated relative to CXCR5- CD8 T cells (12, 13, 15). T cell specific deletion of Id2 results in a dramatic expansion of CXCR5+ CD8 T cells (13, 15, 51). Because Id2 is significantly downregulated in CXCR5+ CD8 T cells, the expression of Id2 may block the development of CXCR5+ CD8 T cells during early CD8 T cell activation. Id3 upregulation in CXCR5+ CD8 T cells may restrain CXCR5+ CD8 T cell development, perhaps after initial subset differentiation. Thus, Id2 contains, and Id3 maintains, E2A induction of Bcl6, TCF-1, and CXCR5 to stabilize the CXCR5+ CD8 T cell phenotype (Figure 4).

Early immunological signals that prompt development of a CXCR5+ CD8 T cell population remain in question. Some evidence for specific cytokine and cellular interactions exists but is largely circumstantial via *in vitro* culture assays. *In vitro* cultures of SIV+ CD8 T cells with IL-12, IL-23, and TGFβ promote CXCR5+ CD8 T cell expansion relative to IL-12 or IL-23 alone (14). When cultured with IL-6, CD8 T cells produce IL-21 similar to CD4 T cells cultured with IL-6 (45). However, in CD8 T cells IL-6 induction of IL-21 via STAT3, inhibits IFNγ, and IL-2 production (45), unlike the robust IFNγ responses in LCMV-specific CXCR5+ CD8 T cells. Although, thus far, CXCR5+ CD8 T cells respond to similar stimuli as CD4 Tfh cells (52–54), these stimuli may induce a context specific response in CXCR5+ CD8 T cells that has yet to be carefully resolved.

In the context of chronic antigen, CD4 Tregs control inappropriate self-reactive responses (55). Within the GC, follicular Tregs maintain T-B interactions to promote B cell differentiation (56). Foxp3+ Tregs localize in close proximity to follicular and extrafollicular CXCR5+ CD8 T cells but with higher frequency to extrafollicular CXCR5+ CD8 T cells (31). During low SIV viremia CXCR5+ CD8 T cell frequency negatively correlated with viral load and positively correlated with follicular Tregs. Whereas, in high SIV viremia, the frequency of CXCR5+ CD8 T cells negatively correlated with follicular Tregs. Together, this suggests that Treg control of CXCR5+ CD8 T cells inhibits function rather than development within the GC, and the efficacy of that inhibition likely relates to viral control (24).

CD8 T cells can be found within the GC of several murine models of spontaneous autoimmune disease including in IL-2-deficient and scurfy mutant autoimmune disease. In these mice, both CD4 Tfh and CXCR5+ PD-1+ CD8 T cells are significantly expanded (29). One common feature of these autoimmune models is a defect in functional Tregs. In the absence of functional Tregs or in conditions of high chronic antigen and inflammation, CXCR5+ CD8 T cells have the capacity to expand and maintain robust effector function by cytokine secretion or direct B cell interactions.

## Conclusions

CXCR5+ CD8 T cells have been found under a number of pathogenic conditions with varied functional capacity. CXCR5+ CD8 T cells promote cell lysis in viral infection and in some cancers, while in inflammation and autoimmunity CXCR5+ CD8 T cells function as helper cells, thus promoting disease pathogenesis. The presence of CD8 T cells within the B cell zone, in combination with their cytolytic and helper functionality, provides the potential for unique interactions with CD4 Tfh cells, B cells and follicular dendritic cells and access to infected CD4 T cells and cancerous B cells that have yet to be fully explored.

Treatments to influence effector responses require a clear analysis of CXCR5+ CD8 T cell function in multiple immune settings that facilitate specific cell interactions. Engineering CD8 T cells to express CXCR5 promotes migration to the B cell follicle (57). While the use of bispecific antibodies optimizes CXCR5+ CD8 T cell targeting of HIV-infected cells via cell specific lysis (17). A combination therapy to optimize CXCR5+ CD8 T cell responses in a patient specific manner will address challenges currently identified in immune non-responding patients to existing HIV treatments. CXCR5+ CD8 T cell activities within and near the follicle provide clues about the immune response that may explain class-switch choices, the development of broadly neutralizing antibodies, and promote a paradigm shift in the nuances of GC reactions.

## Author Contributions

KMV conceptualization, literature evaluation, original draft writing, generated and visualized figures. KKH conceptualization, writing and review, visualization, funding acquisition, and supervision.

### Conflict of Interest Statement

The authors declare that the research was conducted in the absence of any commercial or financial relationships that could be construed as a potential conflict of interest.
